# Endogenous Retroviruses Unveiled: A Comprehensive Review of Inflammatory Signaling/Senescence-Related Pathways and Therapeutic Strategies

**DOI:** 10.14336/AD.2024.0123-1

**Published:** 2024-05-14

**Authors:** Xin-ran Zhao, Jia-bin Zong, Yu-xiao Liu, Tuersun Aili, Min Qiu, Jie-hong Wu, Bo Hu

**Affiliations:** Department of Neurology, Union Hospital, Tongji Medical College, Huazhong University of Science and Technology, Wuhan 430022, China.

**Keywords:** endogenous retrovirus, inflammation, senescence, treatment

## Abstract

Endogenous retroviruses (ERVs), a subset of genomic transposable elements (TEs) in a broader sense, have remained latent within mammalian genomes for tens of millions of years. These genetic elements are typically in a silenced state due to stringent regulatory mechanisms. However, under specific conditions, they can become activated, triggering inflammatory responses through diverse mechanisms. This activation has been shown to play a potential role in various neurological disorders, tumors, and cellular senescence. Consequently, the regulation of ERV expression through various methods holds promise for clinical applications in disease treatment. ERVs also engage in interactions with a variety of exogenous viruses, thereby influencing the outcomes of viral infectious diseases. This article comprehensively reviews the pathogenic cascade of ERVs, encompassing activation, inflammation, associated diseases, senescence, and interplay with viruses. Additionally, it outlines therapeutic strategies targeting ERVs with the aim of offering novel research directions for understanding the relationship between ERVs and diseases, along with corresponding treatment modalities.

## 1. Introduction

**Figure 1.**
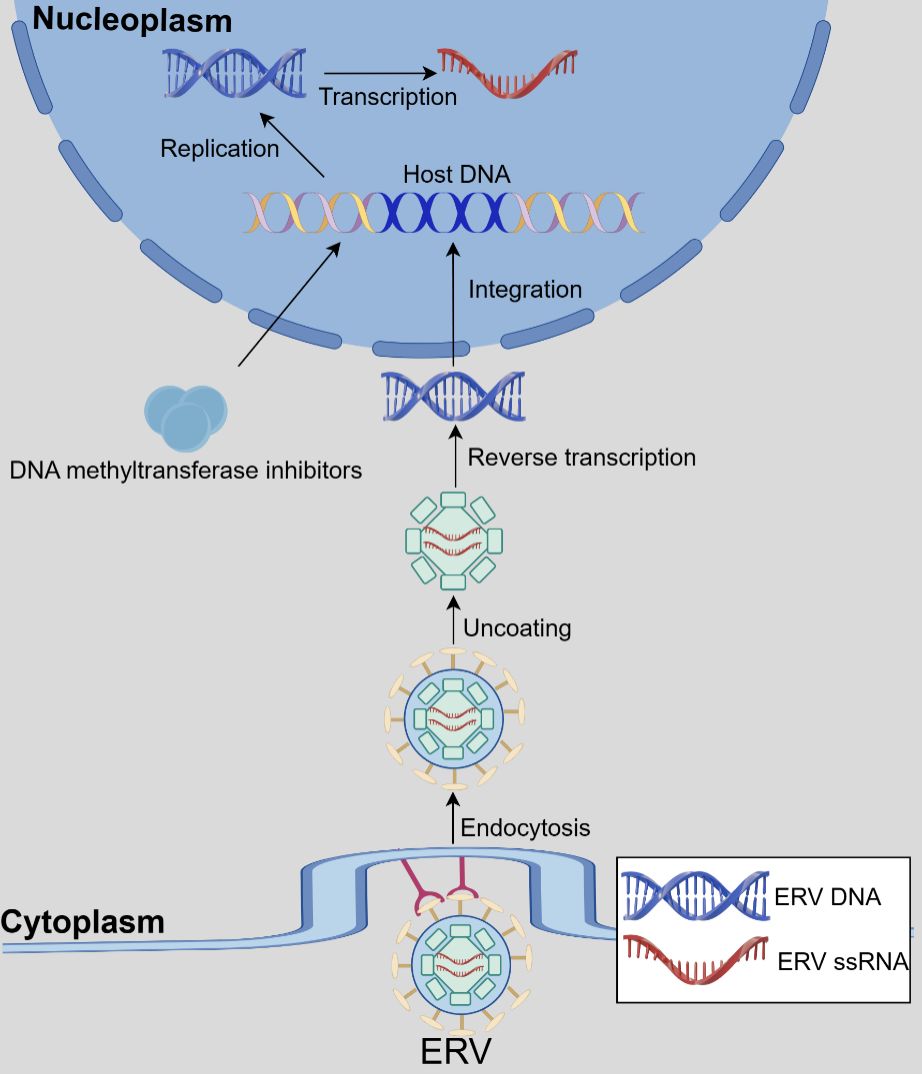
**The endogenization of ERV**. The process involves endocytosis, uncoating, reverse transcription, and integration. DNMTI can induce the expression of ERV. This figure was created by Figdraw.

Over millions of years of evolutionary history, numerous ancient exogenous retroviruses, now extinct, successfully infiltrated host organisms by integrating their DNA into the host cell's genome [1-3] (Fig. 1). In cases where the infected cells are germ cells, the integrated provirus is transmitted to offspring through processes such as replication and reinfection, becoming a permanent genomic feature. Conversely, in cases where the infected cells are somatic cells, the provirus eventually disappears from the genome upon the host’s death [2, 4, 5]. Throughout evolution, human endogenous retroviruses (HERVs) have accumulated extensive inactivating mutations within their coding sequences, resulting in the majority being unable to encode functional proteins due to replication defects [5-7]. Notably, more intact and functional ERVs have been identified in other animals, such as mice and cats. This disparity may be attributed to HERVs being more ancient than ERVs and consequently harboring more defects [7]. Approximately 8% of the human genome consists of HERVs [2, 8].

Over recent years, a significant number of studies have demonstrated that various exogenous factors can activate HERVs, such as antitumor drugs, dietary elements, aging processes, and infectious agents. Part of the rationale behind this activation stems from exogenous factors’ influence on epigenetic regulatory processes [9-11]. Activated HERVs produce specific DNA, RNA, or other intermediates that can be recognized by the host immune system, subsequently triggering an inflammatory response [12-14]. In light of these findings, multiple investigations have established an association between HERVs and various diseases, such as cancer, Alzheimer's disease (AD), schizophrenia, and multiple sclerosis (MS) [12-16]. Targeted therapeutic drugs aimed at HERVs may offer potential benefits to patients with these conditions. HERV also interacts with exogenous viruses, participating in the body's antiviral immune response. In this review, we summarize the modes of HERV activation. Subsequently, we explore the pathways through which HERVs induce inflammation and senescence, as well as its interaction with exogenous viruses. Finally, we discuss the associated pathological outcomes and therapeutic approaches targeting HERVs.

## 2. Activation of ERVs

The typical HERV genome structure comprises gag, pol, and env genes, along with two long terminal repeat (LTR) sequences typically located at both termini. The gag gene encodes the capsid protein, the pol gene encodes the enzymes essential for virus replication and infection, and the env gene encodes the envelope glycoprotein [7, 17, 18]. LTRs harbor promoter and enhancer sequences, which serve as crucial targets for various endogenous and exogenous regulatory factors of ERVs [19, 20]. The sequence similarity of HERVs enables their classification into well-defined subclasses, including HERV-K, HERV-W, HERV-H, and HERV-E [21, 22]. Among these subclasses, the HERV-K family of retroviruses is relatively recent in the context of evolutionary terms and remarkably conserved, with the potential to produce retroviral particles [7, 23, 24]. While most HERV genomes exhibit defects, components encoded by various viral sources within the same cell can recombine to form functional retroviral particles—a process known as trans-complementation [7, 25]. Host immune surveillance tightly regulates HERVs, primarily through epigenetic mechanisms.

### 2.1 Epigenetic regulation of ERVs and their associated activators

DNA methylation, the most widely investigated epigenetic regulatory mechanism governing genomic DNA, extends its influence on the regulation of ERV expression. DNA methylation is believed to inhibit the expression of genomic DNA. By the action of DNA methyltransferases (DNMTs), which transfer a methyl group from S-adenosylmethionine to the fifth carbon atom of cytosine residues within CpG islands, 5-methylcytosine formation. DNMTs are pivotal in embryonic development, with their expression diminishing upon of terminal cell differentiation [26]. However, a study demonstrated that the HERV-K genome exhibits DNA hypomethylation from the eight-cell stage of embryonic development to the emergence of outer blastocyst trophectoderm cells. This hypomethylation augments HERV-K expression, underscoring its significance in embryonic development [27]. This phenomenon may extend beyond HERV-K, such as syncytin, another envelope protein encoded by HERV-W, also plays crucial roles in placental development. Decreased syncytin expression can lead to pregnancy-induced hypertension [28, 29]. The application of DNMT inhibitors (DNMTis) has been shown to foster the expression of HERVs [11, 13, 30, 31].

Histone methylation constitutes another form of methylation, occurring at various histone sites capable of either activating or silencing gene transcription, thereby maintaining gene expression stability [32]. H3K9me3, a frequently observed repressive methylation modification, triggers the silencing of murine ERV expression, requiring the involvement of the SET Domain Bifurcated Histone Lysine Methyltransferase 1 (SETDB1) [33]. A crucial ERV transcriptional regulatory factor is Trim28 (KRAB-associated protein 1, KAP1), which interacts with KRAB-zinc finger proteins (KZFPs) and suppresses human and murine embryonic ERV expression by recruiting SETDB1 [34-36]. Notably, studies have elucidated that the loss of the ZKSCAN3 protein (a type of KZFP) leads to heterochromatin erosion, subsequently inducing cell senescence by activating HERV expression [37, 38]. Further details regarding the mechanism by which ERV induces cell senescence are provided later in this text. Histone acetylation modifications serve to enhance HERV expression. Histone acetyltransferases and histone deacetylases (HDACs) represent pivotal regulatory factors in this context. The combined application of HDAC inhibitors (HDACis) and DNMTis significantly activates HERV expression [39]. However, relying solely on HDACis appears insufficient to trigger HERV expression [40], indicating that histone acetylation may not be as important as methylation in ERV regulation.

Non-coding RNAs, which lack the capacity to encode proteins, function as regulatory RNAs. Numerous studies have elucidated their pivotal role in the regulation of ERV expression [41, 42]. Small interfering RNAs (siRNAs) form silencing complexes through binding with mammalian Argonaute protein 2 (Ago2), thereby resulting in the degradation of target mRNA [43, 44]. Paula et al. disrupted siRNA function by knocking out the *Ago2* gene in mice, subsequently observing an upregulation in ERV expression [44]. PIWI-interacting RNAs (piRNAs) are dispersed in clusters throughout the genome, predominantly operating within germ cells. Their fundamental function lies in the recognition and silencing of specific ERVs to uphold the integrity of genetic material in germline cells [41, 42]. Any disruption in piRNA activity can lead to human infertility [45].

### 2.2 Exogenous activators of ERVs

Various exogenous factors, including high-fat diets, X-rays, ultraviolet radiation, and infections, can induce the expression of ERVs [10, 46-55]. X-rays, ultraviolet radiation, and other forms of ionizing radiation can induce HERV expression by perturbing genomic structure or influencing epigenetic processes, potentially compromising genome stability [46, 56]. Researchers also observed that high-fat diets induced significant skin inflammation with elevated ERV expression in keratinocytes compared to normal diets in mice exposed to Staphylococcus epidermidis [10]. High-fat diets and aging significantly increased the expression of miRNA, lncRNA, and ERV-L in the livers of mice [54]. Furthermore, Shioda discovered that tributyltin (TBT), known to induce obesity, could upregulated ERV expression in testicular somatic cells [55]. Despite numerous observations, the precise mechanisms underlying the elevated expression of ERV in response to a high-fat diet remain incompletely understood.

Exogenous pathogens that can induce ERV expression are various viruses and microbiota. Pattern recognition receptors (PRRs) including cyclic GMP-AMP synthase (cGAS), toll-like receptors (TLRs), retinoic acid-inducible gene protein 1 (RIG-1), and melanoma differentiation factor 5 (MDA5), are pivotal in this phenomenon [49, 51, 52]. These receptors can recognize pathogen-associated molecular patterns (PAMPs) and trigger the release of pro-inflammatory signaling molecules through intricate signaling pathways [57]. Research indicated that germ-free (GF) mice exhibit significantly reduced ERV expression compared to specific pathogen-free (SPF) mice, suggesting the reliance of ERV expression on the gut microbiota. In SPF mice harboring the Myd88^-/-^ mutation, ERV expression is significantly diminished. Conversely, GF mice with the Myd88^-/-^ mutation exhibit a partial restoration of ERV expression [49]. Another study demonstrated that TLR7^-/-^ mice exhibit spontaneous high expression of ERVs and retroviral viremia [58]. Exogenous pathogens can induce inflammation directly through TLR activation. Additionally, they can increase ERV expression by activating TLRs. Subsequently, heightened ERV expression triggers an inflammatory response through the activation of TLRs and other PRRs, thereby amplifying the inflammatory cascade. A comprehensive examination of the mechanisms through which ERV expression induces inflammation is provided in subsequent sections.

## 3. ERVs and inflammation

Following activation by various stimulators, ERVs exhibit overexpression. The resulting DNA, RNA, and proteins serve as PAMPs and are recognized by PRRs, subsequently initiating inflammation through specific pathways. This constitutes the fundamental pathological mechanism of ERVs across various diseases (Table 1). Furthermore, the HERV-K Env protein exerts direct neurotoxic effects. Co-culturing of HERV-K with neurons results in neuronal death, shortened neurites, and decreased neuronal electrical activity. This elucidates the potential mechanism by which HERV-K contributes to the onset of amyotrophic lateral sclerosis (ALS) [59] (Table 1). In addition, recent studies have highlighted the role of ERVs in facilitating the aggregation and intercellular transmission of pathogenic proteins to promote ALS and AD [60] (Table 1).

**Table 1:** Diseases caused by ERVs and associated pathways.

Viral subgroup	Inducible factors	Pathways	Disease	References
**HERV-K**	RNA	TLRs	AD	[12]
**HERV-K**	dUTPase protein	TLRs, RIG-1	Pulmonary arterial hypertension	[91, 206, 207]
**HERV-W**	Env protein (syncytin-1)	TLRs	MS	[15, 90, 208]
**HERV-W**	Env protein (syncytin-1)	TLRs, cGAS-STING	Schizophrenia	[72, 84]
**HERV-K**	RNA	MDA5/RIG-1	Renal fibrosis	[61]
**Murine ERV**	DNA	cGAS-STING	Anxiety/depression	[73]
**HERV-K**	Env protein	Neurotoxic effect	ALS	[59]
**Murine ERV** **HERV-K** **HERV-W**	Env/Gag/Pol protein	Aggregation and dissemination of pathogenic proteins	ALS/AD	[60]
**Various HERV loci**	Ultraviolet radiation	MDA5/RIG-1	Systemic lupus erythematosus	[56]
**HERV-W**	Env protein (syncytin-1)	TLRs	T1 diabetes	[177, 209]

### 3.1 cGAS- stimulator of interferon genes (STING) pathway

cGAS is categorized as a cytoplasmic nucleic acid sensor, primarily responsible for detecting viral or bacterial DNA within the cytoplasm [61-63]. cGAS catalyzes the synthesis of cyclic GMP-AMP (cGAMP) by utilizing ATP and GTP as substrates. Subsequently, cGAMP activates STING. STING, an endoplasmic reticulum protein, phosphorylates interferon regulatory factor 3 (IRF3), facilitating its translocation into the nucleus and its binding with nuclear factor kappa B (NF-κB). This cascade promotes the expression of type I interferons (IFN-I) and inflammatory factors [64-67]. The cGAS-STING pathway is implicated in various neurodegenerative diseases, autoimmune disorders, infections, and tumors [68-72].

**Figure 2.**
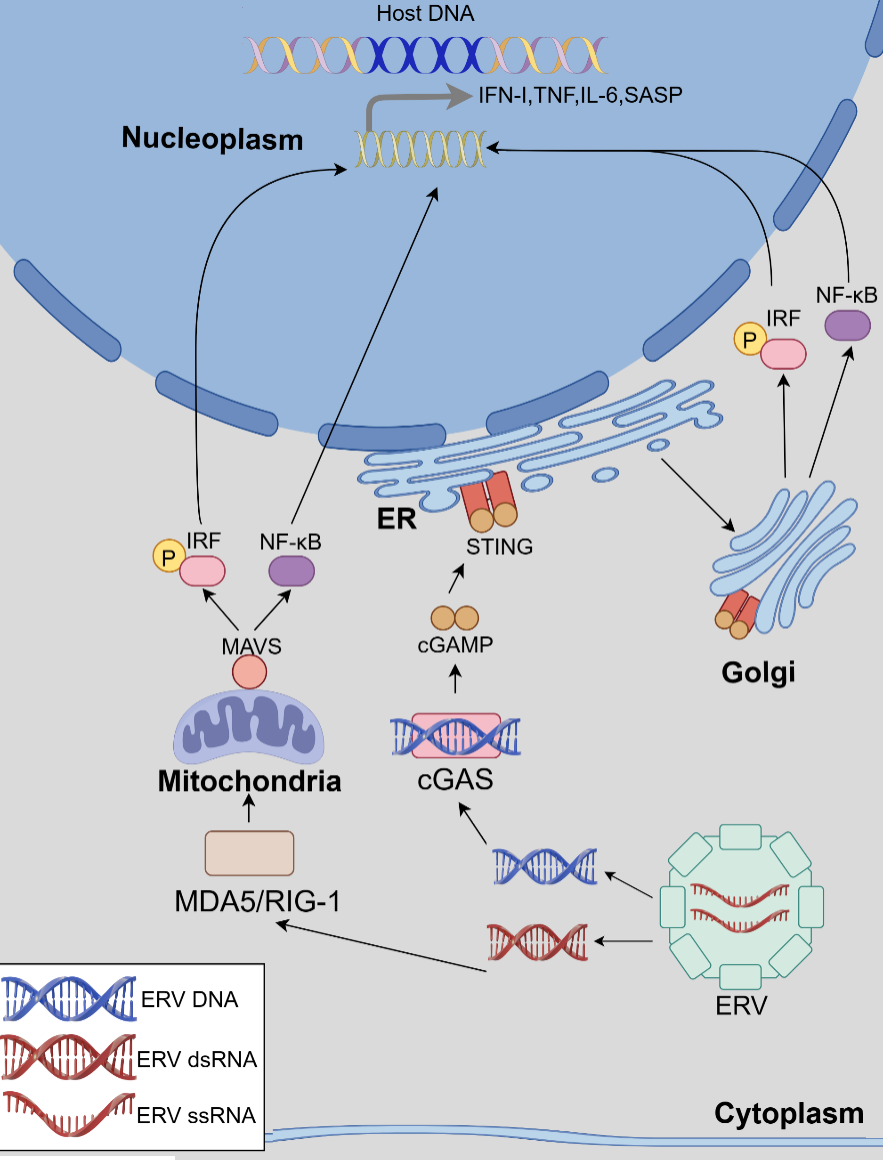
**ERV nucleic acids induce inflammatory responses through cGAS-STING and MDA5/RIG-1-MAVS pathways**. A. cGAS-STING pathway: The intracellular sensor cGAS recognizes ERV-DNA and facilitates the production of cGAMP by utilizing ATP and GTP as substrates. cGAMP, functioning as a secondary messenger, triggers the activation of STING. STING is initially situated in the endoplasmic reticulum and subsequently relocates to the Golgi apparatus upon activation. In this process, STING mediates the phosphorylation of IRF and activates NF-κB, guiding them into the nucleus to modulate the expression of diverse inflammatory factors including IFN. B. MDA5/RIG-1-MAVS pathway: Upon recognition of ERV-dsRNA by MDA5/RIG-1, it translocates to the mitochondria and interacts with MAVS through its CARD domain, subsequently activating MAVS. MAVS, in turn, facilitates the phosphorylation of IRF and the activation of NF-κB. ER, endoplasmic reticulum; Golgi, Golgi apparatus. This figure was created by Figdraw.

Following activation by various environmental stimuli, ERVs activate cGAS through reverse transcription, as demonstrated by multiple studies [10, 73, 74] (Fig. 2). In a study involving stressed mice displaying symptoms of anxiety and depressive-like behaviors, researchers observed significant transcription of mouse intracellular ERV genes MuERV-L, MusD and IAP, as well as activation of the cGAS-STING pathway. Administrating of antiretroviral drugs or knocking down the murine ERV transcription regulatory gene P53 significantly inhibited ERV transcription and the ensuing inflammatory pathway activation, leading to improved behavioral patterns in the mice [73]. Meanwhile, overexpression of HERV can also activate the cGAS-STING pathway. Li et al. observed elevated expression of the HERV-W Env protein in patients with schizophrenia. This heightened expression stimulates IFN-β expression and neuronal apoptosis through the cGAS-STING pathway, establishing a direct link between the HERV-W Env protein and schizophrenia [72].

On the other hand, Yasmine Belkaid et al. reported that the skin microbiota induced the expression of multiple murine ERV to trigger local T cells recruitment through the cGAS-STING pathway. Employing antiretroviral therapy and cGAS/STING knockout both led to decreased ligand DNA production and a significant reduction in the local T cell population [10]. Similarly, a high-fat diet is thought to upregulate murine ERV expression and promote inflammation through the cGAS-STING-dependent pathway [10]. Notably, intracellular DNA sensors may lack specificity, as both damaged DNA and leaked mitochondrial DNA in the cytoplasm can activate them, subsequently triggering an inflammatory response. This process does not involve reverse transcription and is not susceptible to inhibition by reverse transcriptase inhibitors [75, 76].

### 3.2 Inflammatory response mediated by TLRs

TLRs can be classified into several subtypes, with TLR3, TLR7, TLR8, and TLR9 serving as intracellular receptors. TLR3 primarily recognizes double-stranded RNA (dsRNA), while TLR7 and TLR8 predominantly identify single-stranded RNA. TLR9 is the sole sensor capable of recognizing DNA, particularly unmethylated DNA abundant in CpG motifs. Conversely, TLR1, TLR2, TLR4, TLR5, TLR6, and TLR10 are classified as extracellular receptors [77-80].

**Figure 3.**
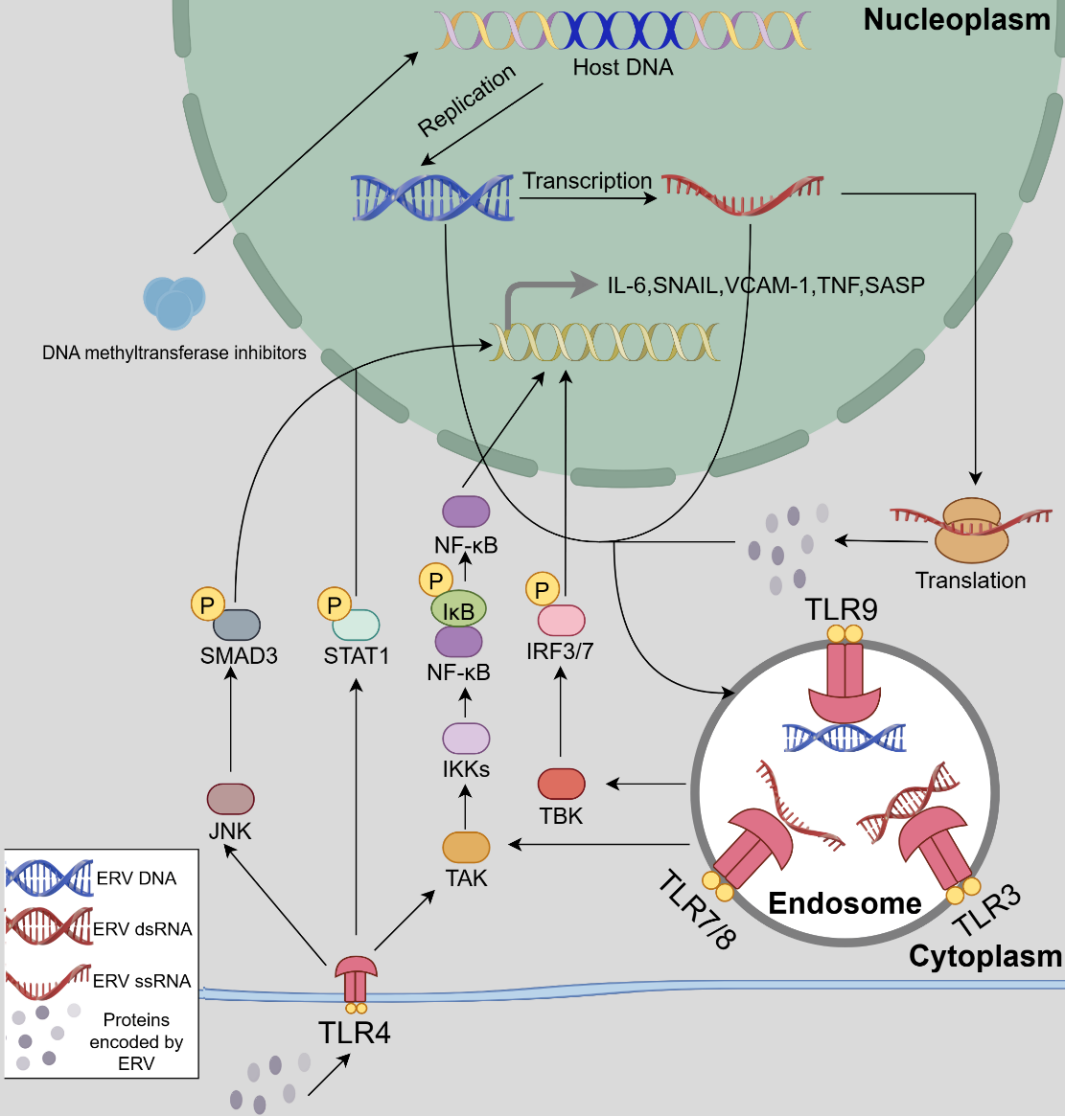
**ERV nucleic acids induce inflammatory responses through the TLRs associated pathways**. Endogenous and exogenous nucleic acids and proteins derived from ERVs have the capability to stimulate TLRs. TLR9 mainly recognizes ERV-DNA, TLR7/8 primarily identifies ERV-ssRNA, TLR3 targets ERV-dsRNA, and TLR4 mainly responds to ERV-related proteins such as HERV-dUTPase. Upon activation by their specific ligands, Toll-like receptors (TLRs) phosphorylate SMAD3 and IRF3/7 via JNK and TBK pathways. Additionally, they phosphorylate the NF-κB inhibitor IκB through TAK-IKKs, leading to its dissociation from NF-κB. These phosphorylated factors and NF-κB cooperatively promote the expression of inflammatory factors such as IL-6. This figure was created by Figdraw.

HERV genes and their transcripts have the capacity to activate both intracellular and extracellular receptors [12, 13, 81] (Fig. 3). Upon extracellular HERV particle infiltration into cells, the RNA produced during their replication process can trigger the activation of TLR7/8. Additionally, HERVs can be activated by various intracellular factors, leading to the generation of RNA that activates intracellular TLRs [12, 13, 81]. For example, a study elucidated that co-cultivation HERV-K with diverse cell types leads to TLR7 activation in mouse astrocytes, macrophages, and neurons, as well as TLR7/8 activation in human neurons and macrophages through the NF-κB-dependent pathway. This activation subsequently stimulates the secretion of various pro-inflammatory factors, potentially contributing to neuronal degeneration and the onset of AD [12]. Furthermore, the utilization of 5-Azacytidine (5-AZA), a DNMTi, can induce HERV activation in human ovarian cancer cell lines, leading to the production of dsRNA capable of activating TLR3. This, in turn, leads to the phosphorylation of IRF3 and IRF7. Phosphorylated IRF3 and IRF7 then translocate to the nucleus, facilitating the expression of IFN-related genes [13, 82].

In addition to HERV-related nucleic acids, HERV-produced proteins can activate TLRs [14, 83-86]. Notably, considerable research has focused on the HERV-W Env protein, also known as syncytin-1 [14, 83, 84]. For example, HERV-W Env has been shown to upregulate TLR4 expression and activate TLR4, leading to tumor necrosis factor α (TNF-α) and interleukin-10 (IL-10) expression [14]. It may also induce nitric oxide (NO) production through inducible nitric oxide synthase (iNOS), potentially causing myelin sheath damage [83]. In another study, Xiao et al. demonstrated HERV-W Env’s role in promoting NO production in microglia via iNOS, though they did not implicate TLRs in this process [87]. In a study on schizophrenia, researchers noted significantly elevated syncytin-1 levels in patients, inducing IL-6 expression and ultimately generating C-reactive protein by activating TLR3 in microglial cells [84]. Another extensively studied HERV-W family virus is the MS-associated retrovirus (MSRV), capable of forming complete infectious viral particles and likely well-conserved throughout evolution [88]. The MSRV Env protein shares significant sequence similarity (94% mRNA sequence similarity) with syncytin-1 [88]. Similarly, the MSRV Env protein exhibits neurotoxic and pro-inflammatory effects, activating TLR4 and inducing the production of inflammatory mediators such as TNF-α, IL-1, IL-6, IL-12p40, and IFN-γ [85, 86, 89, 90], which may damage oligodendrocytes and contribute to MS development. Researchers have also identified another HERV-K synthesized protein, deoxyuridine triphosphate nucleotidohydrolase (dUTPase), when overexpressed, induces inflammation within cells and neighboring cells through vesicular release [91-93]. Primarily activating TLR2/4, dUTPase triggers the expression of cytokines such as IL-6, vascular cell adhesion molecule 1 (VCAM-1), SNAIL, TNF-α, IL-23, and IL-12p40 [91-93]. The authors have detailed the pathways leading to IL-6, SNAIL, and VCAM-1 production in their published work. Specifically, they elucidated that TLR4 activation promotes the expression of these cellular factors through the NF-κB pathway. Furthermore, IL-6 expression requires p-STAT1, SNAIL expression requires p-SMAD3, and VCAM1 expression requires ATF2, mediated by melanoma cell adhesion molecule (MCAM) and its downstream p-ERK [91] (Fig. 3).

### 3.3 Inflammatory response mediated by MDA5 and RIG-1

MDA5 and RIG-1 are members of the PRR family RIG-I-like receptor (RLR), responsible for recognizing viral RNAs during immune responses. These receptors share common structural elements, including an ATPase domain housing DExD/H-box domains in their central region, a C-terminal domain facilitating RNA binding, and an N-terminal caspase activation and recruitment (CARD) domain. The former two structural components primarily facilitate RNA recognition and binding [94-96]. MDA5 predominantly recognizes long-stranded dsRNA, while RIG-1 primarily recognizes short-stranded dsRNA [57]. Upon the activation of these receptors, their CARD domains interact with the mitochondrial antiviral signaling protein (MAVS), thereby initiating NF-κB activation, inducing the production of IRFs, and ultimately leading to the production of IFNs [96] (Fig. 2).

In human ovarian cancer cells, colorectal cancer cells, and renal tubular cells, the utilization of DNMTis, such as 5-AZA, elicits the activation of HERVs, akin to TLR activation. The transcription of HERVs generates dsRNA, which in turn triggers the activation of MDA5 and RIG-1, subsequently instigating IFN responses through the aforementioned pathways [11, 61, 97]. The resultant inflammatory response presents an avenue for utilizing DNMTis as antitumor drugs; however, they may potentially lead to certain diseases [11, 56, 61]. Researchers have observed elevated expression of ERV in both human and mouse kidney samples with chronic kidney disease. Elevated ERV triggers interferon production and kidney fibrosis via the RIG-1/STING pathway. Knockout of RIG-1 or STING in mice demonstrates a mitigating effect on kidney inflammation [61]. Exposure of immortalized human keratinocytes to ultraviolet radiation can induce expression of 165 HERV loci to instigate inflammation through the MDA5/RIG-1 pathway, resulting in keratinocyte apoptosis and inhibition of proliferation. This mechanism could potentially contribute to the pathogenesis of systemic lupus erythematosus [56].

## 4. ERV-induced cellular senescence

Aging is defined as the progressive degenerative changes occurring in cells, organs, and the entire organism with advancing age. Cellular aging lays the groundwork for the organismal aging. The role of chronic inflammation in the aging process is of paramount significance [98, 99]. Unlike acute inflammation, persistent low-level sterile inflammation not only causes to tissue damage and fibrosis but also compromises the immune system, contributing to immune aging [99, 100]. The extended lifespan of women relative to men can be partly attributed to the heightened inflammatory activity observed in older men [101, 102]. In instances of aging driven by chronic inflammation, the aging of hematopoietic stem cells (HSCs) assumes a central role [100]. Aging HSCs secrete immune cells with abnormal functionality, thereby giving rise to the senescence-associated secretory phenotype (SASP). SASPs consist of various factors, including IL-6, IL-1, CXCL-4, CXCL-5, CXCL-6, CXCL-12, CCL-2, CCL-3, CCL-7, CCL-8, transforming growth factor-β (TGF-β), and matrix metalloproteinases (MMPs) [100, 103]. SASPs actively promote cellular senescence, ultimately leading to cell death. Moreover, cellular senescence and cell death release SASPs, establishing a detrimental feedback loop constituting a complex senescence regulatory network [100]. Aging HSCs exhibit an increased inclination toward myeloid cell differentiation and a diminished tendency toward lymphoid cell differentiation [104]. The inflammatory milieu induced by various SASPs further exacerbates this process [105, 106]. Senescent immune cells primarily exhibit the following characteristics: 1. Neutrophils: Reduced resistance to infection, increased apoptosis, and migratory abnormalities attributed to the downregulation of CXCR2 [107-110]. 2. Monocytes/macrophages: Diminished ability to clear senescent cells and upregulation of the genes *p16^INK4A^/pRB* and *p53/p21^WAF1/CIP1^*, mediating cell cycle arrest [111, 112]. 3. Natural killer cells (NK cells): Increased in number, but decreased killing capacity [113, 114]. 4. B cells: Decrease in overall numbers but a relative increase in memory B cells. This shift leads to reduced production of antibodies and plasma cells [115, 116]. Dysfunctional B cells may also contribute to the development of autoimmune diseases [117]. 5. T cells: CD4^+^ T cell numbers decrease more than CD8^+^ T cells. Additionally, there is an increase in Th and Treg cell numbers, but their cytokine secretion decreases. Furthermore, there is upregulation of TGF-β receptor 3 expression on naive CD4 cells, an increase in CD57^+^ T cell proportion, and a loss of CD27/CD28 expression on memory T cells. These changes impair immune function and result in increased SASP secretion [100, 118-121].

In terms of the relationship between ERV and aging, several studies have demonstrated an upregulation in ERV expression levels during the aging process in diverse organisms, including human, non-human primates, mice, drosophila, and yeast [122-124]. Studies involving humans have revealed elevated expression levels of HERV-K and HERV-W in peripheral blood mononuclear cells and red blood cells among older adults [125-127]. Meanwhile, it has been reported that senescent human fibroblasts and mesenchymal stem cells in vitro exhibit upregulated HERVs and corresponding protein products [24, 128]. Notably, Stephen et al. discovered a notable expression of HERVs in human hematopoietic stem and progenitor cells during aging. The authors posit that this phenomenon might contribute to the immune system's clearance of senescent cells [129]. As mentioned previously, the aging of HSCs plays a critical role in human cell aging, emphasizing the importance of HERVs in this process.

Recently, Liu et al. made a noteworthy finding that underscores the association between HERV-K and cellular aging. Their study revealed that overexpression induced by hypomethylation of the HERV-K promoter in human mesenchymal stem cells leads to cellular senescence. This senescence is possibly attributed to HERV-K DNA activating the cGAS-STING pathway, resulting in inflammation and the production of SASPs. Additionally, retrovirus-like particles produced by HERV-K can induce senescence in other cells [24]. The group also elucidated that ERV triggered the production of SASPs through activation of the cGAS-STING pathway, resulting in senescence of the frontal lobe in non-human primates [38].

Take together, researches on the relationship between ERV and aging encompass diverse cell types spanning multiple human systems, such as peripheral blood cells, neurons, immune cells, fibroblasts, mesenchymal stem cells, hematopoietic stem and progenitor cells. This suggests that HERV-induced aging may be widespread. Future studies should be directed at exploring whether targeting HERVs could retard overall aging progression and treat various age-related diseases [130].

## 5. Interaction between ERVs and viruses

Initially, researchers discovered that HERV-K encoded Env and Gag to participate in the assembly of human teratocarcinoma-derived virus particles. This discovery marked the first association of HERV with viral infection [131, 132]. Recent studies have revealed that infections caused by human immunodeficiency virus (HIV), influenza A virus, hepatitis C virus, herpes simplex virus, and the severe acute respiratory syndrome virus 2 (SARS-CoV-2) can induce the expression of HERV [52, 133-135]. For instance, it was reported that patients infected with SARS-CoV-2 exhibited distinct patterns of HERV expression in different disease stages [133]. Till now, our understanding of the interaction between HIV and HERV is profound, whereas our knowledge regarding the relationship between HERV and other viruses remains limited. So, we provide a detailed description of the interaction between HIV and HERV.

### 5.1 Expression of HERV in HIV Infection

Studies have revealed elevated levels of HERV-K RNA in blood monocytes and CD4^+^ T cells in patients with HIV infection [53, 136]. In vitro experiments have also confirmed a significant upregulation of HERV-K expression in HIV-infected cells [137]. Moreover, HIV patients undergoing highly active antiretroviral therapy with notable treatment outcomes exhibit markedly reduced levels of plasma HERV-K RNA compared to those with less favorable responses [138, 139]. The mechanism behind this phenomenon may be attributed to the direct inhibition of HERV-K transcription by antiretroviral medications or indirectly through the suppression of HIV. Researchers have identified that HIV can encode a Tat protein that binds to the LTR sequences of HERV-K, thereby modulating its expression [140-142]. RNA transcriptome sequencing reveals that the Tat protein significantly enhances the expression of 26 unique HERV-K proviruses [141]. Additionally, HIV encodes Vif protein to degrade the host restriction factor APOBEC3G, consequently facilitating the expression of HERV-K [143, 144].

### 5.2 The role of HERV in the anti-HIV virus response

The involvement of HERV in the host's response to HIV infection presents both advantages and disadvantages. On one hand, overexpression of HERV-K stimulates the production of antibodies against the HERV-K transmembrane protein and specific T cells. These antibodies and T cells have the capability to specifically target and eradicate HIV-infected cells, thereby restricting the dissemination of HIV infection [134, 145, 146]. As previously discussed, the overexpression of HERV can activate the expression of inflammatory factors through pathways such as cGAS-STING, TLRs and MDA5/RIG-1. Coincidentally, all these inflammatory pathways are also involved in the immune response against HIV [147-149].

Conversely, proteins encoded by HERV may interact with HIV in a complementary manner. For instance, HERV-W Env can co-assemble with defective HIV viruses, enhancing their infectivity and leading to the formation of pseudo-viruses. These pseudo-viruses can infect CD4^-^ cells, thereby expanding the scope of HIV infection [150, 151]. The protease K10 encoded by HERV-K can be exploited by HIV and exhibits resistance to various protease inhibitors (PIs), including ritonavir and indinavir, potentially contributing to the development of drug-resistant phenomena of HIV infection [152]. While HIV does not directly target neurons, it may induce an upregulation of HERV within neurons. Some HERV proteins have neurotoxic effects, which could contribute to the development of HIV-associated dementia [59, 153].

## 6. Treatment measures directed at ERVs

### 6.1 Epigenetic drugs for cancer treatment

In general, tumors are characterized by global genomic hypomethylation, leading to the selective expression of proteins in patients with cancer, such as cancer-testis antigens (CTAs) in patients with cancer, a phenomenon not observed in healthy individuals. Additionally, patients with tumors demonstrate hypermethylation of specific CpG islands, primarily suppressing the expression of tumor suppressor genes [154-157]. Conventional understanding suggests that DNMTis exert anticancer effects by reversing the suppression of tumor suppressor genes through their demethylating activity. DNMTis, such as azacytidine and decitabine (5-aza-2'-deoxycytidine), have received approval from the U.S. Food and Drug Administration for treating myelodysplastic syndromes and acute myeloid leukemia (AML) [158, 159]. Recent studies have shown that DNMTis activate HERVs through demethylation, triggering antitumor immune responses in a process termed viral mimicry, which holds potential for tumor therapy [11, 160]. Low-dose 5-AZA specifically targets human colorectal cancer-initiating cells by inducing the expression of HERV-derived dsRNA. Activation of the MDA5/MAVS pathway by dsRNA, coupled with subsequent IRF7 induction, likely contributes to the antitumor effect [11]. Another study suggests that DNMTis induce the generation of HERV-derived dsRNA in human ovarian cancer cells, triggering an IFN response through the TLR3 and MAVS pathways, ultimately aiding in the limitation of tumor cells [13].

In addition to fostering the development of a localized antitumor inflammatory milieu, DNMTis can also direct T cells to exert cytotoxic effects by influencing tumor-specific antigens. As previously discussed, DNMTis induces the expression of CTAs through demethylation, serving as tumor-specific antigens. Decitabine, for instance, has been demonstrated to induce the expression of the specific antigen MAGE-1 (MAGE Family Member A1) in melanoma cell lines, leading to their lysis by corresponding cytotoxic T cells [161]. Research involving the HL-60 AML cell line and the T24 transitional cell carcinoma cell line revealed that both azacitidine and decitabine robustly induce the expression of CTAs in these cell lines [162]. Certain HERV-encoded proteins exhibit tumor antigenic properties, such as HERV-E Env protein, which is notably expressed in clear cell renal cell carcinoma (ccRCC). This protein is situated on the surface of tumor cells and directly facilitates the cytotoxicity of immune cells against malignancies [163, 164]. Due to the substantial number and polymorphism of integrated HERVs in the genome, identifying HERVs that can be targeted by CD8^+^ T cells individually is a relatively challenging and time-consuming task. Recently, Bonaventura et al. employed machine learning methods to screen for HERV antigens targeted by CD8^+^ T cells across five types of cancers: colon adenocarcinoma, lung squamous cell carcinoma, head and neck squamous cell carcinoma, lung adenocarcinoma, and bladder urothelial carcinoma, significantly expanding our comprehension of HERVs as cancer antigens [165].

The combined utilization of DNMTis and other epigenetic modifiers demonstrates a heightened potential for inducing ERV expression, necessitating the implementation of appropriate combinatorial therapeutic strategies. Studies have shown the synergistic efficacy of co-administering DNMTis and HDACis in inhibiting the growth of prostate cancer, pancreatic cancer, breast cancer, and ovarian cancer cells. Additionally, this co-treatment facilitates apoptosis and arrests the cell cycle in these malignancies [166-168]. Ashish et al. jointly employed HDACis and DNMTis, identifying thousands of HERV-related novel polyadenylated transcripts (TINPATs) resulting from this treatment. They demonstrated that certain TINPATs function as antigens, triggering T cell responses that target and eliminate cancer cells. These TINPATs were observed in patients with AML undergoing decitabine treatment [169]. Furthermore, Mehdipour et al. identified Alu dsRNA, a reverse repetitive sequence, as the primary source of these immunogenic transcripts. Enhancing the efficacy of epigenetic modifiers can be achieved by preventing the degradation of Alu dsRNA by the enzyme ADAR1, accomplished through the use of ADAR1 inhibitors [170]. In another study, Kogan et al. demonstrated that for AML tumor cell lines characterized by TP53 mutations associated with a dismal prognosis, decitabine alone could induce HERV expression in a STING-dependent manner, concurrently triggering the production of inflammatory factors such as IFN and TNF-α. Conversely, for wild-type AML tumor cell lines devoid of TP53 mutations, a combination of decitabine and poly ADP-ribose polymerase inhibitors (PARPis) achieved similar effects, a feat that decitabine alone could not replicate [160]. The TP53 mutation serves as an adverse prognostic indicator in diffuse large B-cell lymphoma (DLBCL), conferring resistance to conventional therapies such as chemotherapy. Studies have demonstrated that TP53 mutation upregulates the H3K9 methyltransferase SUV39H1, leading to the inhibition of HERV expression via histone methylation [171, 172]. Decitabine administration can counteract this mechanism, enhancing HERV expression, stimulating interferon release, and slowing down tumor advancement [172, 173]. Various approaches to combined drug administration are presented in Table 2.

**Table 2:** Various strategies involving the utilization of epigenetic regulators combined with other drugs.

Treatment A (Name/classification)	Treatment B (Name/classification)	Target	Cancer type	References
**DAC/DNMTi**	TSA/HDACi	Estrogen receptor β	Prostate cancer	[166]
**DAC/DNMTi**	TSA/HDACi	Proteasome, caspase, P53, Ras	Pancreatic cancer	[167]
**DAC/DNMTi**	SAHA/HDACi	ARHI, PEG3	Ovarian cancer	[168]
**DAC/DNMTi**	Pracinostat/HDACi	ERV TINPATs	Multiple cancer cell lines	[169]
**DAC/DNMTi**	Vitamin C	ERV expression	Multiple cancer cell lines	[210]
**DAC/DNMTi**	PARPi	ERV expression	AML	[160]
**DAC/DNMTi**	R-CHOP	ERV expression	DLBCL	[172, 173]

DAC, Decitabine; TSA, Trichostatin A; SAHA, Vorinostat; R-CHOP: rituximab, cyclophosphamide, doxorubicin, vincristine, and prednisone

### 5.2 Monoclonal antibodies (mAbs) targeting ERVs

mAbs primarily exert therapeutic effects by neutralizing pathogenic ERV Env proteins. Currently, the most promising mAb for application is GNbAC1 (Temelimab), the anti-HERV-W Env mAb [16, 174-176]. Temelimab inhibits the interaction between HERV-W Env and TLR4, thereby suppressing the release of inflammatory factors such as TNF-α. It also prevents oligodendrocyte damage, promotes central nervous system myelin regeneration, and reverses insulin secretion inhibition in pancreatic β cells caused by HERV-W Env, thus demonstrating therapeutic efficacy in MS and type 1 diabetes [177-180]. In a double-blind phase II clinical trial for type 1 diabetes, Temelimab reduced the frequency of hypoglycemic events and lowered levels of anti-insulin antibodies. Furthermore, there were no significant differences in the frequency and severity of adverse reactions observed between the treatment and control groups (NCT03179423) [176]. In another phase II double-blind clinical trial utilizing Temelimab to treat patients with MS, the 18 mg/kg dose group exhibited a significant reduction in the number of T1 hypointense lesions in brain magnetic resonance imaging compared to the control group. While there was a statistically non-significant decrease in brain atrophy and magnetization transfer ratio, no serious adverse reactions specifically linked to the treatment were observed (NCT02782858, NCT0323 9860) [181]. Considering the pathogenic role of HERV-W Env protein in schizophrenia, Temelimab holds potential for blocking the production of inflammatory cytokines and neuronal apoptosis induced by HERV-W Env protein through the cGAS-STING and TLR pathways [72, 84]. Additionally, it may regulate the activity of dopaminergic neurons in the brain [182]. However, there are currently no clinical trials to validate the therapeutic effect of Temelimab in schizophrenia. Another specific mAb, K01, targeting the HERV-K Env protein, has demonstrated clinical potential in the treatment of patients with ALS [59], exhibiting a protective effect on both in vitro cultured neurons from patients with ALS and primary motor cortex neurons in mouse brains [59].

In the preceding passage, we discussed the role of specific ERV proteins as tumor antigens. Antibodies targeting these antigens exert a cytotoxic effect on tumors, as evidenced in cases of lung adenocarcinoma [183]. The antitumor activity extends beyond lung adenocarcinoma, research has revealed a significant elevation in serum levels of the HERV-K Env protein in patients with breast cancer, particularly in those with highly aggressive forms of the disease [184, 185]. The application of the anti-HERV-K Env protein mAb 6H5 has demonstrated significant efficacy in suppressing the growth of xenograft breast tumors in murine models [184]. Despite these findings, currently, no monoclonal antibodies targeting tumor-associated HERV antigens are being evaluated in clinical trials. Of note, Barisic et al. isolated CD8^+^ T cell clones specific to the hla-a11-restricted 10-mer peptide antigen (an HERV-E antigen specific to ccRCC) from the peripheral blood of patients with ccRCC and transplanted them into a mouse model. The findings demonstrated that human HERV-E T cells could induce regression of human ccRCC tumor grafts and significantly extend the survival of mice [186]. A phase I clinical trial utilizing CD8^+^ T cells carrying HERV-E TCR for treating ccRCC is presently ongoing (NCT03354390). Furthermore, self-antibodies against HERV-K Env have been detected in the blood of patients with certain autoimmune diseases, such as rheumatoid arthritis and systemic lupus erythematosus. Monitoring the concentration of these antibodies may offer valuable insights into disease progression [187, 188]. A summary of mAbs with potential clinical utility in disease treatment is presented in Table 3.

**Table 3:** mAbs and antiretroviral drugs that exhibit potential clinical utility in disease treatment.

Drug classification	Drug name	Target	Disease	References
**mAb**	Temelimab	HERV-W Env	MS	[179-181]
**mAb**	Temelimab	HERV-W Env	T1 diabetes	[176, 178]
**mAb**	Temelimab	HERV-W Env	CIDP	[175]
**mAb**	Temelimab	HERV-W Env	Schizophrenia	[72, 84, 182]
**mAb**	K01	HERV-K Env	ALS	[59]
**mAb**	6H5	HERV-K Env	Breast cancer	[184]
**Combination antiretroviral drugs**	Triumeq	HERV-K Env	ALS	[192, 193]
**NNRTI**	Efavirenz	HERV-W Env	MS	[194]
**Integrase inhibitor**	Raltegravir	HERV-W Env	MS	[196]
**PI**	Lopinavir/Ritonavir/ Azanavir	HERV-K	Schwannoma Meningioma	[198]
**NNRTI**	Efavirenz	HERV family	ASD	[211]
**NRTI**	Tenofovir/Emtricitabine/ Lamivudine	Murine ERV	Anxiety/depression	[73]

CIDP, Chronic inflammatory demyelinating polyneuropathy; ASD, Autism spectrum disorder; Triumeq, Abacavir, Dolutegravir, and Lamivudine.

### 6.3 Antiretroviral drugs targeting ERVs

Owing to the genomic structural similarities between HERVs and HIV, antiretroviral drugs designed to target HIV are also effective against HERVs [189]. However, the efficacy of these drugs varies depending on the specific virus subtype and the class of antiretroviral drug employed. Research indicates that nucleoside analog reverse-transcriptase inhibitors (NRTIs) such as zidovudine, stavudine, didanosine, lamivudine, and tenofovir, as well as non-nucleoside reverse-transcriptase inhibitors (NNRTIs) such as efavirenz, exert inhibitory effects on HERV-K103. In contrast, other NNRTIs, PIs, and integrase inhibitors do not impact HERV-K103 [190], as supported by studies that Towler et al. observed the resistance of HERV-K to various HIV PIs in their findings [152]. Tyagi et al. further elucidated that NRTIs combining with the integrase inhibitor raltegravir effectively inhibited HERV-K replication and infection, while the blocking effect of PIs was relatively weak [191]. These findings support the prevailing notion that NRTIs generally exhibit effectiveness against various retroviruses, whereas PIs, NNRTIs, and integrase inhibitors are tailored specifically to combat HIV [190]. In summary, NRTIs appear efficacious in curtailing HERV-K replication, while the effects of NNRTIs, PIs, and integrase inhibitors remain debatable. High HERV-K expression is implicated in ALS. In a phase II clinical trial utilizing Triumeq (composed of abacavir, dolutegravir, and lamivudine) for ALS treatment, results indicated that this medication could decrease the levels of HERV-K in the serum of patients, alleviate ALS progression, and did not result in significant adverse reactions (NCT02868580) [192, 193].

Initially, researchers observed a decreased incidence of MS among individuals with HIV compared to the general population. This occurrence could be attributed to the antiretroviral treatment administered to patients with HIV, which may exert therapeutic or preventive effects on MS [194, 195]. MS is associated with high expression of HERV-W. Regarding HERV-W, Morandi et al. conducted an in vitro experiment revealing that among several drugs such as lamivudine (NRTI), tenofovir (NRTI), darunavir (PI), efavirenz (NNRTI), and raltegravir (integrase inhibitor), only efavirenz possesses the capability to inhibit HERV-W Env expression [194]. In a phase II clinical trial evaluating raltegravir as a treatment for patients with MS, the findings indicated no impact on the progression of MS lesions or patient outcomes (NCT01767701). This outcome may be attributed to improper drug selection, timing of administration, and dosage of antiretroviral medications [196].

Some endogenous reverse transcriptases are encoded by ERVs, typically suppressed in normal tissues but abnormally active in embryonic and tumor tissues [197]. This phenomenon may be associated with heightened HERV expression in specific tumor tissues. Notably, multiple studies have demonstrated the inhibitory effect of antiretroviral drugs on tumor proliferation [197-201]. For example, HERV-K is overexpressed in Merlin-negative schwannoma and all types of meningiomas, promoting tumor cell proliferation. The use of PIs, such as lopinavir, azanavir, and ritonavir, effectively curbs HERV-K-induced tumor cell proliferation [198]. These findings underscore the clinical potential of antiretroviral drugs in tumor treatment. For a comprehensive list of antiretroviral drugs with potential clinical utility in disease treatment, please refer to Table 3.

## 7. Conclusion and future perspectives

In recent decades, our understanding of ERV has significantly deepened, shedding light on its mechanisms of inappropriate activation and its pivotal role in inflammation. This enhanced comprehension has unveiled a close association between ERV and aging, neurodegenerative diseases, autoimmune diseases, and cancer. The present review provides a multidisciplinary summary of the recent advances in ERV-related research by combining virology, immunology, epigenetics, biology of aging, pathophysiology of age-related diseases, and clinical medicine.

The research on ERVs represents a rapidly advancing frontier, characterized by numerous unresolved questions. HERVs make up approximately 8% of the human genome, with a limited proportion having been causally associated with diseases and aging [202, 203]. Despite the majority being considered non-functional, their persistence throughout evolutionary history raises the question of their potential utility. To adequately address this, more extensive interdisciplinary investigations are required. Recently, some existing studies have primarily established correlations between HERVs and diseases; however, more longitudinal, and mechanistic investigations are crucial for a comprehensive understanding.

Concurrently, various drugs targeting ERV, such as epigenetic modifiers, mAbs, and antiretroviral drugs, are in development and undergoing clinical trials. Indeed, taking the role of mAbs in the treatment of MS as an example, one significant advantage of employing these novel drugs over conventional immunomodulators lies in their lesser interference with the immune system, thereby minimizing the risk of severe immunosuppressive complications, making them suitable for long-term treatment. Nonetheless, a substantial obstacle in the advancement of mAbs, is the inability to directly assess their efficacy and side effects in model organisms due to disparities in HERV sequences and those of other species. Comparative studies have also revealed that the onset of neuroprotection by mAbs generally lags behind that of conventional medications [204]. Future research efforts should not only concentrate on the development of innovative ERV-targeted drugs but also strategically investigate the optimal timing for modulating ERV expression, whether by up-regulation or down-regulation. Furthermore, real-time ERV concentration monitoring holds significant potential in predicting disease progression and prognosis. Presently, combination therapy approaches primarily entail the concurrent use of diverse epigenetic modifiers. In the future, it may be worthwhile to investigate the synergistic effects of combining various classes of ERV-targeting medications.

Finally, our comprehension of the relationship between foreign pathogens and disease is more profound compared to ERVs. These foreign pathogens can exacerbate cardiovascular disease by activating PRRs such as cGAS [205], a mechanism that also underlies the pathogenesis of neurological disorders resulting from ERV activation. To date, no study has reported a direct link between ERVs and cardiovascular diseases. Enhanced investigations of the associations between ERVs and other diseases may provide new therapeutic innovations.
